# Novel *TBX1* loss-of-function mutation causes isolated conotruncal heart defects in Chinese patients without 22q11.2 deletion

**DOI:** 10.1186/1471-2350-15-78

**Published:** 2014-07-06

**Authors:** Yue-Juan Xu, Sun Chen, Jian Zhang, Shao-Hai Fang, Qian-Qian Guo, Jian Wang, Qi-Hua Fu, Fen Li, Rang Xu, Kun Sun

**Affiliations:** 1 Department of Pediatric Cardiology, Xinhua hospital, Affiliated to Shanghai Jiao Tong University School of Medicine, 1665 Kongjiang Road, Shanghai 200092, China; 2 Key Laboratory of Cell Differentiation and Apoptosis of Chinese Ministry of Education, Shanghai Jiao Tong University School of Medicine, Shanghai 200025, China; 3 Medical Laboratory, Shanghai Children’s Medical Center, Affiliated to Shanghai Jiaotong University School of Medicine, Shanghai 200127, China; 4 Department of Pediatric Cardiology, Shanghai Children’s Medical Center, Affiliated to Shanghai Jiaotong University School of Medicine, Shanghai 200127, China; 5 Scientific Research Center, Xinhua hospital, Affiliated to Shanghai Jiao Tong University School of Medicine, 1665 Kongjiang Road, Shanghai 200092, China

**Keywords:** TBX1 haploinsufficiency, 22q11.2 deletion, Conotruncal heart defects, Molecular dynamics simulation

## Abstract

**Background:**

TBX1 and CRKL haploinsufficiency is thought to cause the cardiac phenotype of the 22q11.2 deletion syndrome. However, few unequivocal mutations of *TBX1* and *CRKL* have been discovered in isolated conotrucal heart defects (CTDs) patients. The aim of the study was to screen the mutation of *TBX1* and *CRKL* in isolated CTDs Chinese patients without 22q11.2 deletion and identify the pathomechanism of the missense mutations.

**Methods:**

We enrolled 199 non-22q11.2 deletion patients with CTDs and 139 unrelated healthy controls. Gene sequencing were performed for all of them. The functional data of mutations were obtained by *in vitro* transfection and luciferase experiments and computer modelling.

**Results:**

Screening of the *TBX1* coding sequence identified a *de novo* missense mutation (c.385G → A; p.E129K) and a known polymorphism (c.928G → A; p.G310S). In vitro experiments demonstrate that the TBX1^E129K^ variant almost lost transactivation activity. The TBX1^G310S^ variant seems to affect the interaction of TBX1 with other factors. Computer molecular dynamics simulations showed the *de novo* missense mutation is likely to affect TBX1-DNA interaction. No mutation of *CRKL* gene was found.

**Conclusions:**

These observations suggest that the TBX1 loss-of-function mutation may be involved in the pathogenesis of isolated CTDs. This is the first human missense mutation showing that TBX1 is a candidate causing isolated CTDs in Chinese patients without 22q11.2 deletion.

## Background

Congenital heart defects (CHDs), consisting of heterogeneous anatomy with distinct phenotypic subtypes, are the most common human birth defects worldwide, affecting nearly 0.8% of live births. Of these cases, 15 ~ 20% are CTDs [1]. CTDs are severe malformations associated with cyanosis and many other serious manifestations of hypoxia and are characterised by a disordered orchestration of the ventricle, the aorta and the pulmonary artery. They include tetralogy of Fallot (TOF), double outlet of right ventricle (DORV), pulmonary atresia with ventricular septal defect (PA/VSD), transposition of the great arteries (TGA), interrupted aortic arch (IAA), and persistent truncus arteriosus (PTA), leading to cardiac enlargement, ventricular dysfunction, poor quality of life, and even sudden death in the absence of surgical treatment.

The pathogenesis of CTDs is heterogeneous and involves multiple genes and environmental factors. Although our understanding of molecular pathways in cardiac development has grown remarkably in the past few years, the aetiology of human CHDs cannot be identified or explained in the majority of cases [2]. Caused by a heterozygous multi-gene deletion, 22q11.2 deletion syndrome (22q11DS) is a relatively common genetic disorder (1:4000 live births). CTDs are a prominent part of the 22q11DS phenotype, with a frequency of approximately 75% in 22q11DS patients. Reports originating from Western countries associate 12.8-17.8% of CTDs to the del22q11, and our own work showed a lower incidence (6.13%) in Chinese CTDs patients [3]. The *TBX1* gene, a member of a phylogenetically conserved T-box gene family of DNA-binding transcription factors, is mapped to the 22q11.2, and is hypothesised to be responsible for the cardiac phenotype of 22q11.2 deletion syndrome. Mutations of the *TBX1* gene have been detected in some patients featuring DGS/VCFS who are otherwise devoid of the 22q11.2 deletion [4]. Mutational analysis in nonsyndromic CTDs patients only detected a insertion of 30 bp within exon 9c of *TBX1*, caused polyalanine stretches [5,6]. To better address the issue of the link between *TBX1* and isolated CTDs, we performed *TBX1* gene sequencing in a large sample of Chinese isolated CTDs patients without 22q11.2 deletion.

*CRKL*(*CRK-Like*), another gene located within the 22q11.2 and expressed in the developing heart, was found to be deleted in a few CTDs patients with atypical distal 22q11.2 deletions not including TBX1 [7,8]. The *Crkl*^*-/-*^ mice embryos had DORV, VSD [9,10]. *Crkl* and *Tbx1* genetically interact, and developmental defects associated with loss of *Crkl* and *Tbx1* were linked to two major signalling pathways (RA and Fgf8) during embryonic development in model organisms [10,11]. These lines of evidence led us to hypothesize that mutations of *CRKL* may also be implicated in the pathogenesis of human CTDs. To test this hypothesis, we sequenced the exons and the flanking areas of *CRKL* gene in all CTDs patients in our cohort.

## Methods

### Ethics statement

All the assessments were performed with the approval of the Medical Ethics Committee of Shanghai Children’s Medical Centre (SCMC) and the Medical Ethics Committee of Xinhua hospital. All written informed consent was obtained from the parents of each patient and control.

### Subjects

The patients were recruited prospectively in SCMC from June 2008 to December 2009. The cardiac phenotype was confirmed by angiocardiography and echocardiography in all the patients. When available, the surgical operative notes were reviewed. Of 212 CTDs patients, 199 (78 female, 121 male; median age 2.1 years) were enrolled because they were negative for the 22q11.2 microdeletion when tested by MLPA using the SALSA P250-A1 MLPA-DiGeorge syndrome test kit (MRC-Holland, Amsterdam, The Netherlands) [3]. They included 70 TOF, 50 DORV, 28 PA/VSD, 28 TGA, 4 IAA and 2 PTA patients, as well as 17 other cases of conotruncal malformations (11 single ventricle with malposition of great arteries (SV/MGA), 4 hypoplastic right heart syndrome (HDHS), 2 coarctation of aorta (CoA)). The clinical information of 199 patients was examined by trained clinicians and none of them had extracardiac anomalies and features suggestive of 22q11.2 deletion syndrome. All of the subjects were of Han ethnicity. Parental informed consent was obtained for all of the patients enrolled before blood samples were drawn at the time of catheterisation. A total of 139 unrelated Han Chinese children (57 female, 83 male; median age 7.43 years; all physically and mentally healthy) were included as normal controls. We detected the cardiac morphology of these controls by transthoracic echocardiography and confirmed they were normal. After obtaining informed written parental consent, a peripheral venous blood sample was obtained, and the genomic DNA was extracted from peripheral lymphocytes using a QIAamp DNA Blood Mini Kit according to the manufacturer’s instructions (Qiagen, Duesseldorf, Germany).

### Sequencing

**Figure 1:**
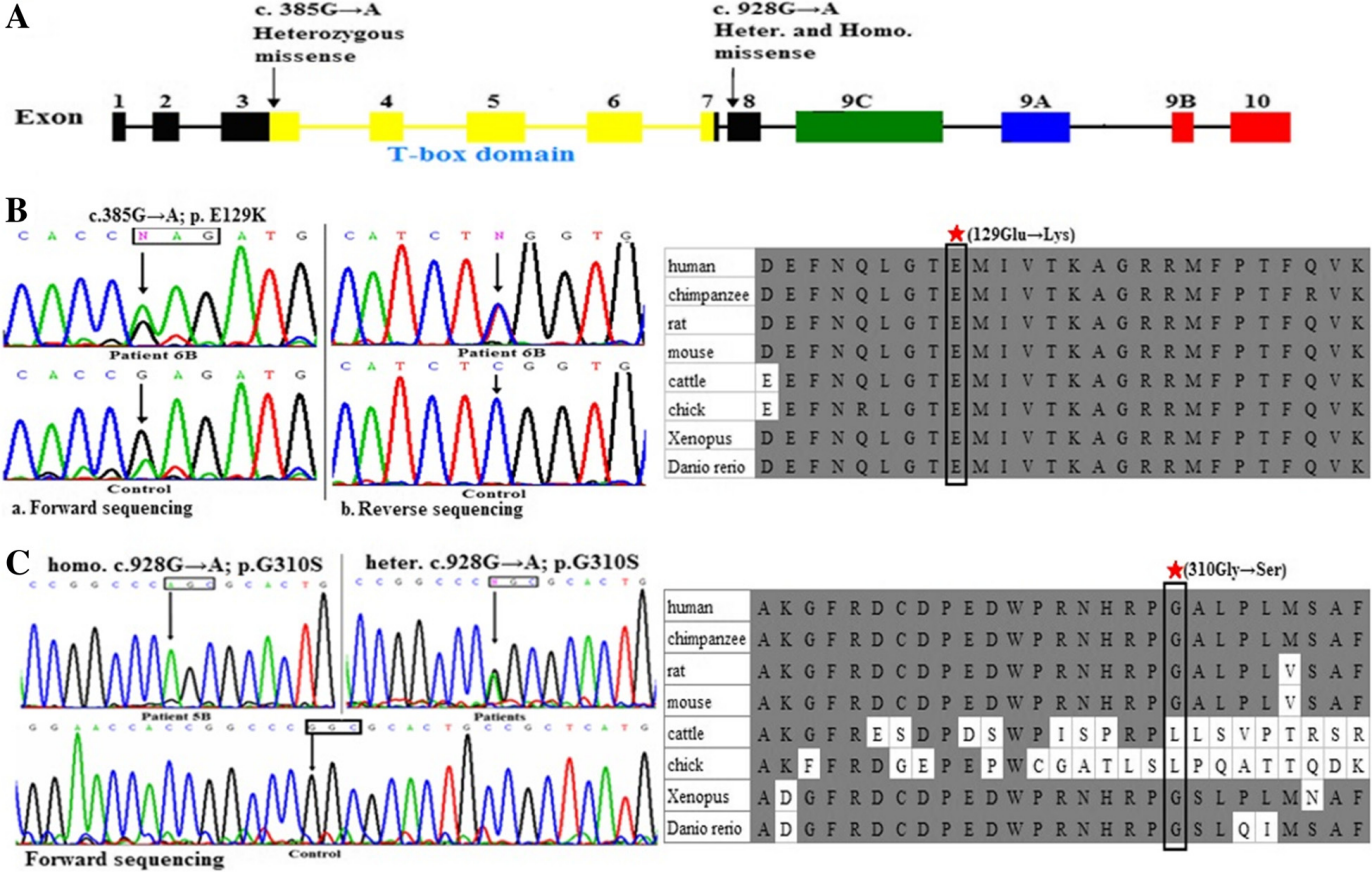
**The screening results of the *TBX1 *coding sequence. (A)** Schematic representation of the TBX1 gene. The conserved T-box domain is marked with yellow squares and spans exons 3-7. The two variants described in this paper are indicated by black arrows. **(B)** Shown at the left are the DNA sequences of the 385G → A mutation (upper panel) and the wild-type *TBX1* sequence (lower panel). The fragment was sequenced from forward and reverse directions. Shown at the right is the homologues sequence alignment of a part of the T-box domain of TBX1 from various vertebrates. **(C)** Shown on the left are the DNA sequences of the *TBX1* gene with a 928G → A change. The three electropherograms show the homozygote AA, heterozygote GA, and homozygote GG. The homologues sequence alignment is shown at the right. The conserved amino acids are shown with a grey background, and residue 129 and 310 is indicated with a black box.

The coding regions of the three alternative spliced forms of the *TBX1A*, *TBX1B*, *TBX1C* (Figure 1A) and *CRKL* were sequenced in the 199 CTDs patients and 139 controls. We designed primers that amplify all exons and flanking intronic sequences. The primers (Table 1) were designed following the corresponding genomic regions available in the Genbank database (TBX1, NG_009229.10; CRKL, NG_016354.1) using Primers3 software (http://bioinfo.ut.ee/primer3/). The PCR was performed with the genomic DNA and the products were sequencing on an ABI 3130 sequencer (Applied Biosystems). The sequence traces were aligned with the reference sequence using the GenBank BLAST program (http://blast.ncbi.nlm.nih.gov/Blast.cgi).

**Table 1:** **Primer sequences used to amplify and sequence all the coding exons of the *TBX1 *** **and *CRKL *** **gene and exon/intron boundaries**

**Primers**	**Orientation**	**Sequences**	**Fragment size**
TBX1-EXON1	F	5’-AGGAGCAGATGTCTCAGCCC-3’	594 bp
	R	5’-CACCGGCTGCCTATACTCAC-3’	
TBX1-EXON2	F	5’-ATGACGCCATAATCCTCTGG-3’	725 bp
	R	5’-TGTGTTTTCTCCCCTTTGCT-3’	
TBX1-EXON3	F	5’-TCACGCAGCTCTCGCATTTC-3’	628 bp
	R	5’-CCGGCGGAGGATAGGTGTTA-3’	
TBX1-EXON4	F	5’-CAAGCTCCCAGTTGAGTAGG-3’	414 bp
	R	5’-GCAGGTGCCTAAAGAGTTTC-3’	
TBX1-EXON5	F	5’-GGCAGCAGAGGGTTCAATCT-3’	457 bp
	R	5’-GCCTCGCAGGGACTCTAAAG-3’	
TBX1-EXON6	F	5’-TGACCCAGCCTCATCTTGGA-3’	404 bp
	R	5’-AGGTCTAAGCGGACCCACTG-3’	
TBX1-EXON7-8	F	5’-GGTGCGCTTCTCCTAACACTC-3’	722 bp
	R	5’-GGAGAGGGCCGAGGAGTG-3’	
TBX1-EXON9C	F	5’-CCAAGAGCCTTCTCTCCGC-3’	775 bp
	R	5’-TGGGGAACCGGATACTTCGA-3’	
TBX1-EXON9A	F	5’-CGTTGGGAGATGCAGTCCT-3’	853 bp
	R	5’-GCTTACTGGACAGCAGCAC-3’	
TBX1-EXON9B	F	5’-CTGATGGTGTGTGAGGCTGA-3’	636 bp
	R	5’-CACCTCTTGCATGCACACTT-3’	
TBX1-EXON10	F	5’-CTGCTCTGTTTGAGGTGGTT-3’	632 bp
	R	5’-ACGCATCAGCTTTTATGGGA-3’	
CRKL-EXON1_1	F	5’- GACGGTGCTCCTGATTGGCT-3’	747 bp
	R	5’-CCCAGGGCAGGTGGAAGAAT-3’	
CRKL-EXON1_2	F	5’-GGACAGCCGCCGCCCCTACC-3’	434 bp
	R	5’-CCCACCCCCCTATACAAGCA-3’	
CRKL-EXON2	F	5’-CAGTGAGCTAAGATCACGCT-3’	677 bp
	R	5’-ATGTCAAAGGACCCAAAAAG-3’	
CRKL-EXON3	F	5’-AAGGATCACTTGAGCCCAGG-3’	376 bp
	R	5’-AGGCAGAACAACAAAGCAGC-3’	

### Plasmid constructs

The *TBX1* expression vector containing the cDNA of human TBX1C was purchased from Origene (Rockville, MD, USA). The *TBX1* cDNA was digested and inserted into the plasmid pcDNA3.1(+) at the KpnI and XhoI sites. The mutant expression vectors were generated by use of site-directed mutagenesis performed according to the protocol provided by the Quickchange Site-Directed Mutagenesis Kit (Stratagene, La Jolla, California, USA), and the wild-type pcDNA3.1-TBX1 was used as the template.

**Figure 2:**
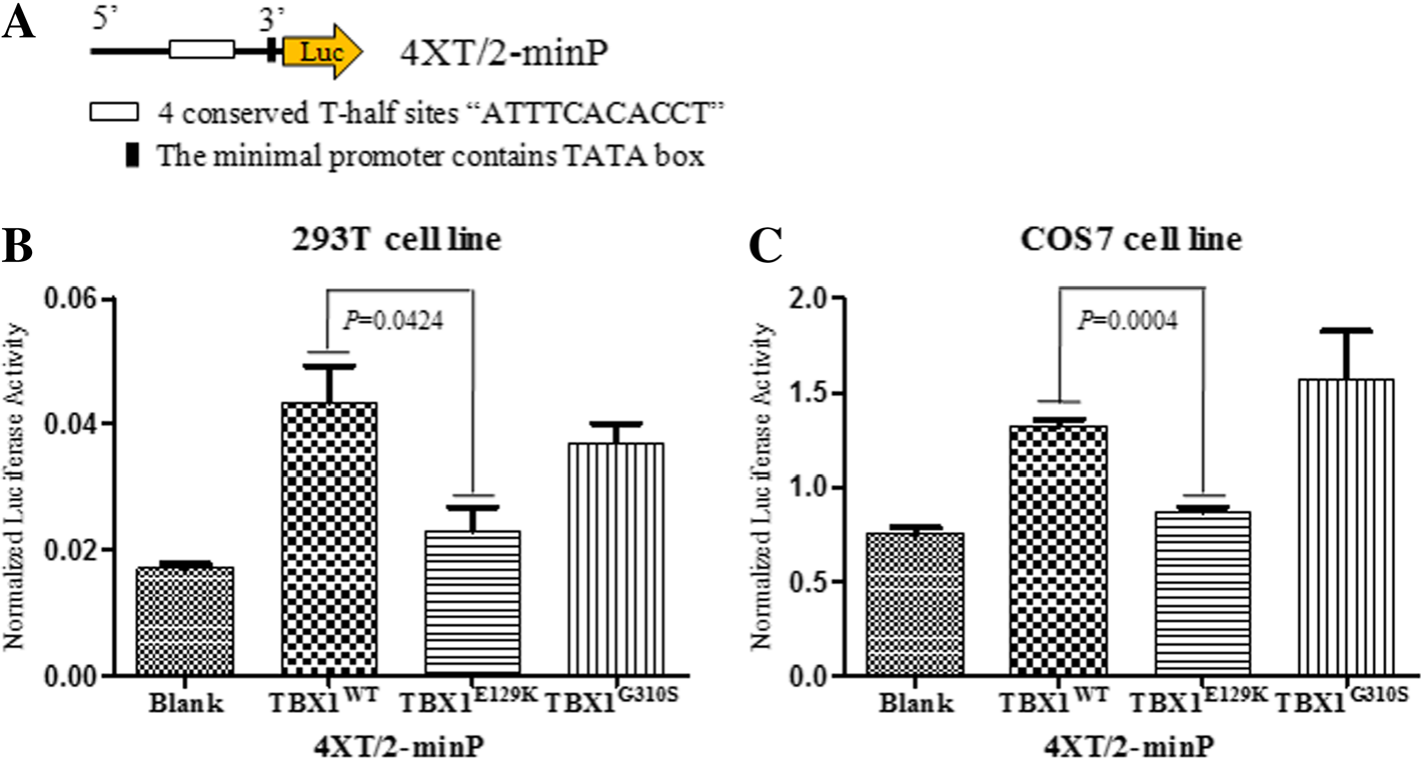
**Transcriptional activation of luciferase reporter constructs by the wild-type and mutant TBX1. (A)** Schematic diagram of 4XT/2-minP reporter construct. Four conserved T-half sites “ATTTCACACCT” were oriented head to tail, synthesised and subcloned into the KpnI − HindIII sites in the pGL4.25 [luc2CP/minP] plasmid. **(B)** and **(C)** show that the 293 T or COS7 cells were co-transfected with the 4XT/2-minP reporter construct containing the T-box binding elements and either a pcDNA3.1 (+) control vector (Blank), the TBX1 wild-type construct, or the mutant constructs. The results were normalized for transfection efficiency to a co-transfected renilla luciferase vector and are shown as the mean ± SEM of three independent experiments performed in triplicate.

Four conserved T-half sites “ATTTCACACCT” were oriented head to tail, similar to those reported by Sinha et al.[12] and were synthesised and subcloned into the KpnI − HindIII sites in the pGL4.25 [luc2CP/minP] plasmid (Promega, Madison, Wisconsin, USA) to generate the 4XT/2-minP reporter construct (Figure 2A). The sequence and orientation of the luciferase reporter were verified by DNA sequencing.

### Transfection and Luciferase assays

The transient transfection was performed with FuGENE HD transfection reagent (Promega, Madison, Wisconsin, USA) according to the manufacturer’s protocol for adherent cells. The HEK293T and COS7 cells were maintained in DMEM medium (Invitrogen, California, USA) with 10% foetal calf serum (Invitrogen, California, USA) and were then co-transfected with 100 ng of the wild-type or mutant TBX1 constructs, 500 ng of the 4XT/2-minP reporter construct, and a renilla luciferase pGL4.74 [hRluc/TK] vector (Promega, Madison, Wisconsin, USA). The cells were harvested 40 h after transfection. The firefly and renilla luciferase activity were measured using the Dual Luciferase Kit (Promega, Madison, Wisconsin, USA) and the Centro XS^3^ LB 960 Microplate Luminometer (Berthold, Bad Wildbad, Germany) according to the manufacturer’s recommended protocol. The activity of the firefly luciferase was normalised to that of renilla luciferase. The results shown are the mean ± s.e.m of three independent experiments performed in triplicate.

### Molecular dynamics (MD) simulations of wild-type and mutant TBX1

Based on the X-ray crystal structures (PDB entry: 4A04, resolution 2.1 Å) [13], the models of TBX1 and its mutant for the MD simulations were built in Sybyl version 6.8 (Tripos Inc., St. Louis, MO). First, the sidechains with missing coordinates were reconstructed using the fragment library of the Biopolymer module; then the modified structures were subjected to energy minimization in the Minimize module using the Powell method up to the gradient tolerance of 0.05 kcal/(mol · Å) to relieve possible steric clashes and overlaps of side chains. The 3D structural model of the mutant TBX1 was built up using the xLeap module in the AMBER suite (version 8.0) [14].

MD simulations were conducted on the two systems of wild-type and mutant TBX1 using the AMBER suite of programs (version 8.0) with the parm99 force field [15]. Each structure was prepared by using the xLeap module in AMBER, in which protons were added to the structure and all inoizable side-chains were maintained in their standard protonation states at pH7.0. A truncated octahedral box of TIP3P waters was added with a 10 Å buffer around the complex and counterions were added to maintain the electroneutrality of the two systems. To avoid the instability that might occur during the MD simulations, the solvated systems were subjected to minimization for 5000 cycles with protein restrained and followed by another 5000 cycles with the whole systems relaxed. Then, the systems were gradually heated from 0 K to 300 K during the first 60 ps by three intervals, followed by equilibrium for 80 ps under constant volume and temperature condition. Afterwards, the systems were switched to constant pressure and temperature condition and equilibrated for 100 ps to adjust the systems to a correct density. Finally, the production simulations were carried out in the absence of any restraint and two 5-ns MD simulations were then conducted on the wild-type and mutant TBX1 to probe the function of the mutation.

All the MD simulations were performed using the parallel version of PMEMD in the AMBER suite. The long-range electrostatic interactions were calculated using the particle mesh Ewald method, whereas the SHAKE algorithm was employed to fix the lengths of the bonds involving hydrogen atoms [16,17]. During the simulations, the integration time step of 2 fs was adopted and structural snapshots were flushed every 500 steps (1 ps). The non-bonded cut-off was set to 10.0 Å, and the non-bonded pair list was updated every 25 steps. By applying the Berendsen algorithm, each production simulation was coupled to a 300 K thermal bath at 1.0 atm pressure [18]. The temperature and pressure coupling constants were set to 2.0 ps and 1.0 ps, respectively [14].

## Results

### Mutation screening of the *TBX1* and *CRKL* gene in CTDs patients without 22q11.2 deletion

Screening of the *TBX1* coding sequence identified a *de novo* variant, as well as one known polymorphism G310S (Figure 1). The *de novo* mutation is localised in exon 3 (c.385G → A; p.E129K) in heterozygosis (Figure 1B). The patient was a 12 years old boy at the time of initial admission to SCMC and suffered from PA/VSD and patent ductus arteriosus (PDA). His height was 152 cm, and weight 50 kg on admission. His appearance looked normal. He didn’t show mental retardation. Due to cardiac problem, he did not finish primary school. Unfortunately he lost the opportunity to operation because of pulmonary hypertension and died at the age of 13. His parents are non-consanguineous marriage. The mutation was not observed in both of his normal parents. The E129K mutation, involving conversion from an acidic amino acid residue (Glu) to an alkaline amino acid residue (Lys), occurs within the evolutionarily conserved T-box domain in the highly conserved residue found in TBX1 orthologues of human, chimpanzee, rat, mouse, cattle, chicken, Xenopus, and Danio rerio (Figure 1B).We also detected a homozygous single nucleotide polymorphism c.928G → A; p.G310S in one male patient with HDHS, TA, VSD, TGA, pulmonary stenosis (PS), and atrial septal defect (ASD). His parents, who were verified to be carriers of heterozygosis, had no cardiac phenotype. The heterozygous variant was detected in 15 CTDs patients and seven normal controls. The G310S is located near the C-terminal of the T-box domain and conserved in the TBX1 vertebrate orthologues of humans, chimpanzees, rats, mice, Xenopus, and Danio rerios, but not in cattle and chickens (Figure 1C).

We sequenced PCR products containing coding exons from *CRKL* using the sanger method, and no mutations were detected.

### Functional analysis of the mutations

To examine further the functional significance of the detected mutations in TBX1, we engineered the c.385G → A (p.E129K) mutation in the human TBX1 cDNA. The non-synonymous SNP c.928G → A (p.G310S) was constructed simultaneously. The polymorphism G310S showed no significant effect on the transcriptional activation in our assay, in contrast to the wild-type TBX1. It exhibited a slight decrease in transactivation relative to the wild-type in the 293 T cell line, yet a slight increase in transactivation in the COS7 cell line. The novel missense mutation E129K showed significantly reduced transcriptional activity in the 293 T and COS7 cells lines in our assay compared with the wild-type protein (*p* < 0.05) (Figure 2B and 2C).

### Molecular models and MD simulations of wild-type and mutant TBX1

**Figure 3:**
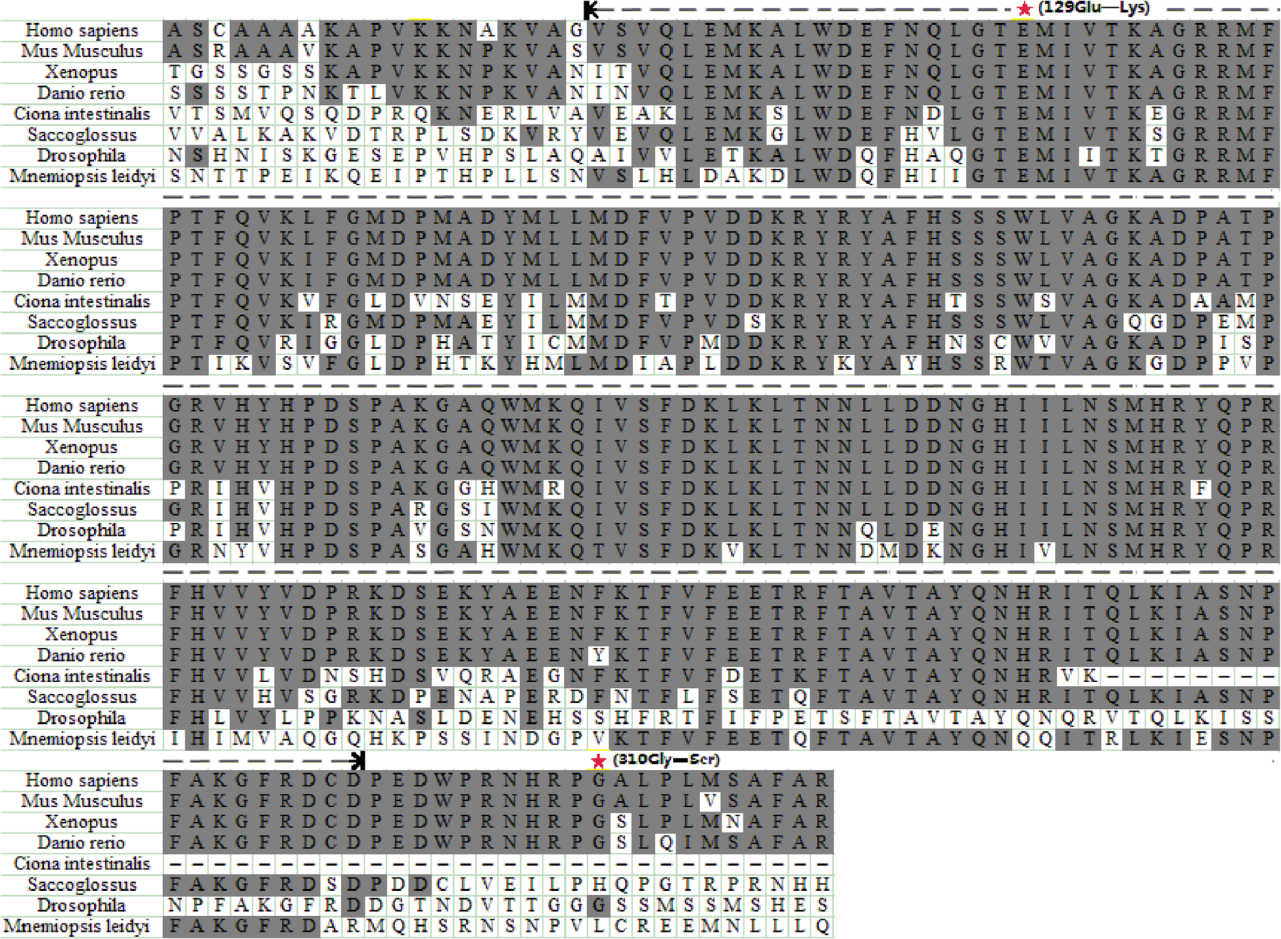
**The phylogenetic sequence alignment of the T-box domain and 20 additional amino acids from different species located at the N and C terminus of the T-box domain.** The phylogenetic analysis is from vertebrate mammals to marine zooplankton. The aminoacid residues that are identical to the TBX1 sequences are shaded. The red pentagram demonstrates the amino acid substitutions of E129K and G310S in the TBX1 found in patients. The dotted line indicated the T-box domain.

**Figure 4:**
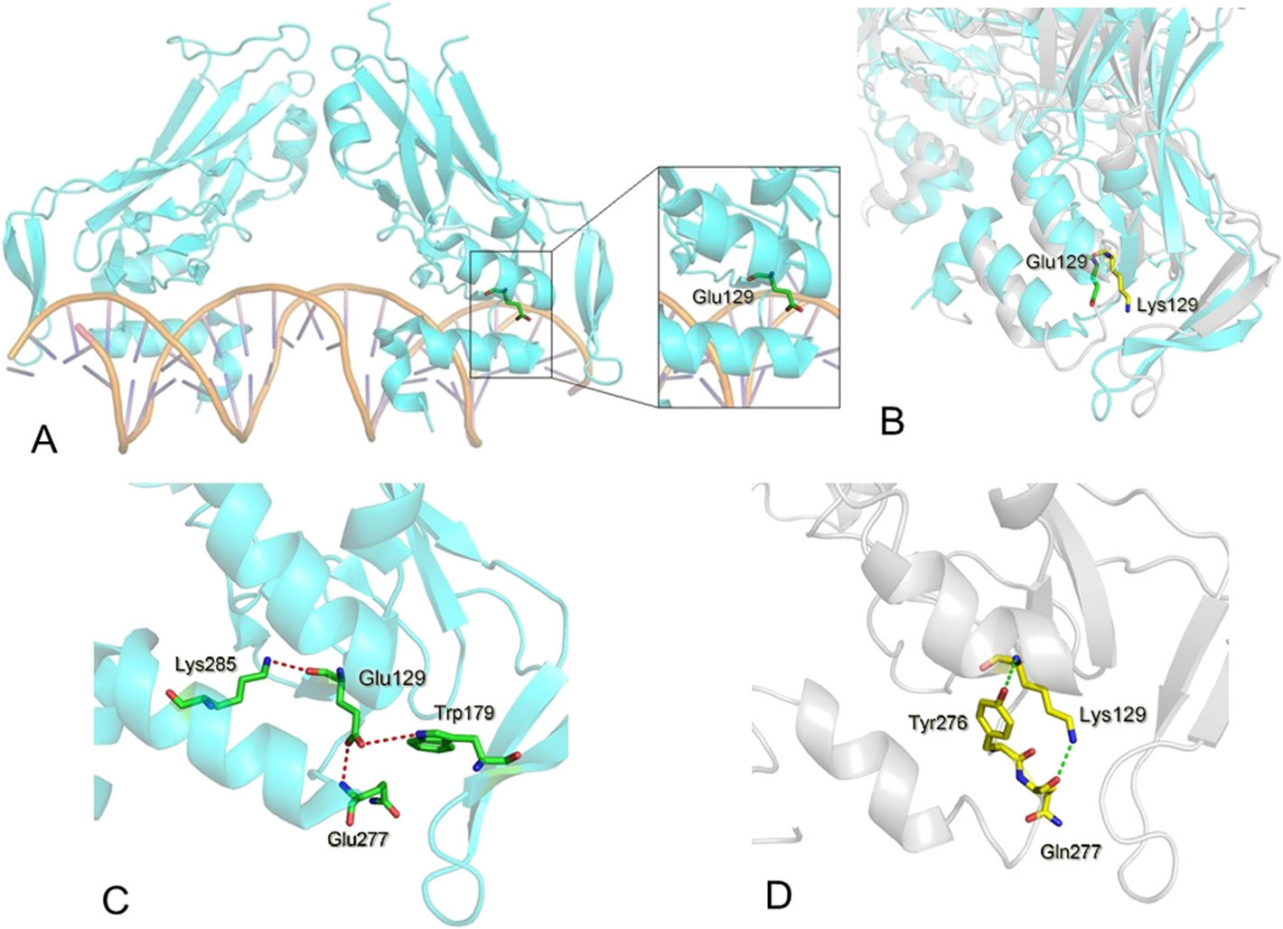
**Schematic diagram of TBX1 bound to DNA. (A)** The wild type TBX1 structure with E129 is shown as sticks. **(B)** The superimposed structures between the wild-type TBX1 (cyan) and the TBX1^E129K^ (grey) and the carbon atoms from E129 and its mutant K129 are colored in green and yellow, respectively, and the conformations are extracted from the end of the 5-ns simulations. **(C)** Local interactions with E129 in the wild-type TBX1. **(D)** Local interactions with K129 in the mutant TBX1. The hydrogen bonds around E129/K129 are shown as dashed lines.

To interpret the effect of the point mutation E129K on the TBX1 function, we modelled the structure of the TBX1 DNA binding domain on the basis of the TBX1 DNA crystal structure (PDB code 4A04) using Sybyl version 6.8 (Tripos Inc. St. Louis. MO) and the structural model of TBX1^E129K^ using the xLeap module in the AMBER suite (version 8.0). Residue glutamic acid 129 (E129) is an evolutionarily conserved residue within the T-box domain (Figure 3). It is clear from the structure that E129, which sits on a beta strand, interacts with tryptophan 179 (W179), glutamine 277 (Q277) and lysine 285 (K285) by forming hydrogen bonds (Figure 4C). Q277 and K285 are located at the helix-turn-helix motif on the C-terminal of the protein, which is involved in recognising the DNA through its minor groove [15]. The terminal negative charges of the side-chain in the E129 acidic residue are replaced with positive charges (K129, alkaline) in the mutant E129K-TBX1 (Figure 4A and B), leading to a complete disruption of the hydrogen bonds between E129 and its surrounding residues (Figure 4C and D) and a distortion of the binding interface between the TBX1 and DNA (Figure 4B), which may have a negative effect on the protein-DNA interaction and thus the associated transcriptional activation/inhibition functions.

## Discussion

The *TBX1* gene is a member of the T-box gene family of transcription factors that is characterised by a conserved DNA binding domain throughout the metazoan evolution [19]. The members of the T-box gene family play a crucial role in a wide variety of developmental processes in vertebrate and invertebrate embryos [20]. The biological importance of these genes is emphasised by the phenotypes of the patient carried loss-of-function mutations of *TBX3* and *TBX5*[21-23].

TBX1 haploinsufficiency has long been known to be crucial in the aetiology of 22q11DS. Mutation analysis has revealed frameshift and missense mutations of *TBX1* in patients with the 22q11DS-phenotype, but no detectable deletion [4,24,25]. Animal model experimental evidence suggests that *Tbx1* is required for normal heart development [26,27]; however, no unequivocal mutation of *TBX1* has been discovered in isolated CTDs patients. Tbx1 is expressed in the secondary heart field (SHF) and positively regulates SHF cell proliferation and contribution to the muscle layer of outflow tract (OFT), of which the myocardium derives from the SHF [28-30]. We investigated a cohort of isolated CTD patients without 22q11.2 deletion and identified one novel mutation (E129K) of TBX1. E129K, located within the conserved T domain, is predicted to affect TBX1-DNA interaction on the basis of the structures of DNA-bound wild-type or mutant TBX1 (Figure 3). This finding is in line with the experimental data showing that in our transient transfection and luciferase reporter assays, the TBX1^E129K^ variant has almost lost transactivation activity for the transcription directed from the consensus palindromic DNA-binding site (Figure 2B and C).

The T-box genes are exquisitely dose-sensitive and often act in a combinatorial or hierarchical fashion [26,31]. The T-box domain, as the basic characteristic of the T-box family, is a DNA-binding region and a protein-protein interaction domain for other transcription factors, chromatin remodelling complexes and histone-modifying enzymes [31]. It has been suggested that Tbx1 interacts with the BAF chromatin remodelling complex and histone methylases to regulate transcription [32]. The known variant, G310S, positioned in a conserved region downstream to the T-box domain, was found in our CTDs patients and healthy controls. The TBX1^G310S^ variant exhibits opposite behaviour in the 293 T and COS7 cell lines. In the 293 T cell line, the variant partially loses the transactivation on reporter with the conserved half-T sites (Figure 2B), whereas it slightly enhances the transactivation in the COS7 cell line (Figure 2C). Prior studies have reported that the TBX1^G310S^ variant prevents TBX1-SMAD1 interaction that could not affect by the mutations that abolish transactivation [33]. We hypothesise that there may be a critical region for protein-protein interaction that resides downstream to the T-box region, and the different behaviour of the TBX1^G310S^ mutant in the 293 T and COS7 cell lines may be caused by the distinct cofactors for TBX1 function in those cells. Although the heterozygous variant was found in the patients and controls, the homozygote was only detected in a patient with complex CHD. We cannot exclude that the TBX1^G310S^ mutant protein might have an effect on the pathogenesis of congenital malformations.

*CRKL* gene lies within the typically 3 Mb deleted region in 22q11DS patients. *CRKL* encodes an SH2-SH3-SH3 adaptor protein, and is part of a signalling pathway that includes *Focal adhesion kinase* (*FAK*) and *MAP kinases* (*ERK1* and *ERK2*) [34]. The embryonic phenotypes of murine neural crest conditional knockout of *Fak* or *Crkl* are similar and show cardiac outflow tract abnormalities [10,11,34]. These experimental observations indicate that the Fak-Crkl-Erk signalling pathway is critical important for cardiac outflow tract development. However, only four patients with CTDs have been reported with atypical distal 22q11.2 deletion that involves *CRKL*, but not *TBX1* so far [8]. Sequencing analysis of the alleles or the remaining allele failed to identify a *CRKL* mutation [8,35]. In our study, *CRKL* was sequenced in 199 CTDs patients without extracardiac anomalies, and we also failed to detect a mutation. We therefore speculate that the coding region mutation of *CRKL* gene may not implicated in the pathogenesis of CTDs.

We recognize some weaknesses that are inherent to our study. We only sequenced the coding region and flanking areas between exons and introns. In genome, non-coding regions (promoter regions and regulatory elements) play an important role on gene expression. Further studies are needed to be carried out to clarify the link between variants in non-coding regions and the pathogenesis of CTDs.

## Conclusions

We have demonstrated that a novel *TBX1* nucleotide sequence variant is a mutation that might be involved in the pathogenesis of isolated CTDs in non-22q11.2 deletion patients, based on the results of sequence alignment and functional analysis of the mutants. The novel TBX1^E129K^ mutant, hypothesised to have loss-of-function status, may cause heart defects by haploinsufficiency. Other than the E129K mutation, the known TBX1^G310S^ variant seems to affect the interaction of TBX1 with other factors.

## Competing interest

The authors declare no conflicts of interest.

## Authors’ contributions

YJX and SC collected the samples, performed most of the experiments, and draft the manuscript. JZ built up the three-dimensional structural model of TBX1 and provided Figure 4. SHX and QQG helped with luciferase experiments. JW help to collect samples and screen the individuals. QHF and FL participated in the design of the study. RX and KS conceived of the study, participated in its design and coordination, analyzed the data and helped draft and revise the manuscript. All authors read and approved the final manuscript.

## Pre-publication history

The pre-publication history for this paper can be accessed here:

http://www.biomedcentral.com/1471-2350/15/78/prepub
